# Students’ Perceptions of Their Student-Operated Restaurant Experience: A Qualitative Study

**DOI:** 10.3390/nu15092186

**Published:** 2023-05-04

**Authors:** Allison Ells Clark, Brittany Woodbury, Emily Vaterlaus Patten, Nathan Stokes

**Affiliations:** Department of Nutrition, Dietetics, and Food Science, College of Life Sciences, Brigham Young University, Provo, UT 84602, USA

**Keywords:** foodservice management education, nutrition education, student-operated restaurant, qualitative

## Abstract

As there is a rapid increase in the consumption of food outside the home, it is essential that future foodservice managers are well prepared for their critical role in menu creation and nutrition care planning in a variety of foodservice establishments. Student-operated restaurants (SORs) are one method of experiential learning used to educate future foodservice managers. This study aimed to explore the students’ perceptions of their SOR experience and the extent to which nutrition concepts were incorporated in their program. This is a research area that has not been explored previously. For this study, a total of eighteen students from four universities were recruited via email to participate in interviews. Results from the qualitative thematic analysis of interview data revealed the following three overarching themes related to students’ SOR experience: (1) Interpersonal Relationships and Mentoring, (2) Assessment of Immediate Experience, and (3) Moving Forward, Added Value, and Growth. Regarding nutrition, while some students felt that nutrition principles were effectively addressed during their SOR experience, other students acknowledged a lack of nutritional emphasis in their SOR and expressed a desire for a greater application of the nutritional principles learned in other classes. The SOR experience described by students was rich as they developed a variety of relationships and skills.

## 1. Introduction

In today’s society, meal consumption outside the home is becoming increasingly more frequent [1,2]. In fact, the portion of Americans’ food budget spent outside the home increased by 9% from 1984 to 2010 and Americans are consuming 23% more of their daily calories away from home in 2012 than in 1978 [3]. These trends are concerning since frequency of eating outside the home is correlated with increased obesity rates and greater risk of all-cause mortality [4,5].

As obesity rates increase, an interest in healthy eating is also rising in the public with consumers increasingly seeking more nutritious options and more specific nutrition information [6]. Despite this interest, chefs do not often take opportunities to improve the nutritional content of menu items due to perceived barriers, such as low consumer demand [7]. The discrepancy between consumer preference and the response of the restaurant industry is highlighted in a study which showed that most culinary students thought nutrition would be an important part of their career, but only one third felt that the restaurant industry valued nutrition as important [8].

Foodservice managers play a crucial role in menu creation and nutrition care planning and may therefore be well prepared to help improve nutritional content of the menu items [9]. Although education among foodservice managers varies widely, the typical certification required to become a foodservice manager is a high school diploma [10]. A higher level of education can be a significant benefit as this position can include a wide range of managerial and nutritional responsibilities [9]. Education, training, and experience widen the scope of professional practice which may increase capacity to expand nutritional opportunities [11].

Experiential learning has the potential for greater effectiveness than a traditional classroom model [12]. When students participate in experience-based learning activities, they understand the course material better and are better able to apply it in their careers. Students engaged in experiential learning also benefit from improved memory of concepts due to first-hand experience. Hands-on experience can also lead to personal insights which shape students’ beliefs about themselves and their learning [13]. Student-Operated Restaurants (SORs) are a mode of experiential learning for foodservice management education that are used in university-level dietetics and/or hospitality programs. Each program has curriculum requirements set by its accrediting foodservice and/or management body [14,15]. SORs provide a setting in which these competencies can be met through hands-on activities, such as preparing and serving food to customers [16].

Previous research has explored different aspects of SORs including the customer experience, the educational experience through the perspective of SOR managers, and the application of nutrition principles by SOR managers [17,18,19]. However, to date, no research has been conducted to examine the viewpoints of students within these programs. The purpose of this study was to understand students’ perceptions of their experiences in SORs, including the role of nutrition in their instruction. The following are the specific objectives:

1. Interview students regarding their perceptions of their overall SOR experience, including nutrition-related practices
2. Assess students’ confidence with basic foodservice competencies
3. Assess students’ confidence with management competencies

## 2. Methods

### 2.1. Study Design

Due to the lack of understanding regarding student perspectives of their SOR experience, qualitative methods were utilized for this study [20,21,22]. Specifically, semi-structured interviews were utilized to guide discussion with participants during interviews and explore various aspects of their experiences.

### 2.2. Recruitment and Distribution

To reach participants who had experienced the phenomenon being studied, purposeful maximum variation sampling was utilized for recruitment [22,23]. A brief recruitment survey was developed to gather information regarding whether students had participated, were currently participating, or were planning to participate in an SOR, at which university they participated, and other basic demographic characteristics. An email with a link to the survey was then sent to five program directors of SORs in the US. The email included a brief description of the study along with the survey link to be forwarded to students. Upon completion of the survey, students were asked to provide an email address to be used when scheduling an interview. Research assistants contacted the students using that email address and scheduled interviews. As compensation, students who participated in the interview were given a USD 25 Amazon gift card. Approval from the university’s Institutional Review Board was received prior to recruitment and data collection.

### 2.3. Interviews

Using the email addresses provided by participants in the demographic survey, researchers contacted each participant and scheduled an interview time. The research team for this project consisted of one white female who is a dietitian with a PhD in Hospitality and Dietetics Administration, one white male with a PhD in Hospitality Management, and two white female undergraduate students. The two research team members with PhDs had extensive prior experience in conducting qualitative interviews as well as qualitative data analysis. Prior to conducting interviews for this study, the undergraduate research team member was trained and were guided to conduct two practice interviews. After each interview one of the experienced research team members provided feedback to the student on how to improve their interview skills. Researchers also discussed and reviewed potential biases prior to data collection. Interviews were conducted independently by the undergraduate student research team member via Zoom and each lasted approximately 30 to 60 min. Prior to starting the interview, participants were given an opportunity to review the informed consent form and their verbal consent to partake in the interview was obtained. An interview guide was used throughout the interview to prompt discussion. This guide included questions about students’ perceptions of SOR experiences, foodservice and management competencies, nutritional influence, and value of the SOR experience to student career goals. Interviews were recorded and the audio recordings were saved and sent to a professional transcription service for transcription.

### 2.4. Data Analysis

Frequencies for the demographic data were calculated using SPSS, v. 28 (IBM Corporation, Armonk, NY, USA). Interview transcripts were analyzed using qualitative thematic analysis methods [24]. First, three researchers independently read through the interview transcripts in order to familiarize themselves with the data. The researchers then met and collaboratively identified three overarching themes drawn from commonalities in the participants’ answers. Using those three themes, researchers then developed a codebook to be used when coding participant transcripts. Next, two researchers independently coded the transcripts for the three themes. The researchers met regularly throughout the process to discuss discrepancies in coding, update the code book, and calculate inter-rater reliability. The overall inter-rater reliability for all transcripts was 88%.

## 3. Results

Participants (n = 18) included in this study were enrolled in dietetics (n = 13) and/or hospitality (n = 5) programs at four universities in the US and had previous (n = 7), current (n = 7), or upcoming (n = 4) experience in SORs. See Table 1 for additional demographic characteristics. Interview responses were analyzed using qualitative thematic analysis methods that resulted in three overarching themes. The themes identified were (1) Interpersonal Relationships and Mentoring, (2) Assessment of Immediate Experience, and (3) Moving Forward, Added Value, and Growth. Illustrative quotes within each theme and sub-theme are provided in Table 2.

### 3.1. Theme 1: Interpersonal Relationships and Mentoring

Within the first theme, Interpersonal Relationships and Mentoring, three sub-themes were identified: working together, relationships, and mentoring. Working together was discussed as an enjoyable and constructive aspect of SORs. Students indicated that working as a team maximized performance and confidence in their tasks. It also fostered communication among different areas of the kitchen. One student said, “[I] feel like it’s a very team-oriented kind of class. We’re all able to work together and help one another” (participant 10, dietetics).

Students found the relationships formed in SORs to be among their most valuable takeaways. They indicated that SORs provided an environment where they could foster closer relationships with classmates compared to other lecture-based classes. One student said, “I thought it was a good way for me to grow closer to the people in my class” (participant 3, dietetics). Participants also discussed relationships they formed with professors, interns, and teaching assistants in SORs. These leaders served as role models by demonstrating and implementing foodservice and management practices alongside the students. One student said, “I think it’s just fun to see we’re at the beginning, they’re at the end. It’s fun to have them as those role models” (participant 6, dietetics).

The third sub-theme was mentoring. Students discussed the different methods of mentoring they experienced in the SORs. Many students noted that they were surprised at how much autonomy they were given by their mentors. Although students were given more independence than they anticipated, most students found this to be an effective mentoring strategy. One student said, “I thought the professors did a good job of letting us figure things out on our own, but also offering us assistance when we needed to, so that we learned how to problem solve” (participant 3, dietetics). Other effective mentoring methods included one-on-one instruction and assistance with problem-solving. A few students reported feeling excessive pressure not to make mistakes. However, most students felt their instructors were understanding of errors and used them as constructive teaching opportunities.

### 3.2. Theme 2: Assessment of Immediate Experience

Within the second theme, Assessment of Immediate Experience, three sub-themes were identified: hands-on learning, time, and nutrition. Many students highlighted hands-on learning as a beneficial aspect of their SOR experience. By experiencing various foodservice roles and rotations, students learned how parts of the foodservice system interrelate. Another benefit of the hands-on element was that it allowed the students to apply their learning from lecture classes. One student said, “I like how it’s super hands-on. So everyday coming to lab and just being able to physically do stuff instead of just sitting at a desk. I like how you get to interact with other people and customers” (participant 17, dietetics). Although most students had a good experience in their SOR, some felt they received no added benefit to their previous foodservice experience.

Time was frequently mentioned in students’ assessment of their SOR experiences. Many felt that it was a considerable time commitment on top of other required classes. They also found their shifts to be long and tiring. One student said, “It feels like I’m taking three classes in one, along with so many other classes that I still have multiple assignments for throughout the week. So that part is overwhelming” (participant 14, hospitality). Other comments centered on the difficulty of completing required tasks in the allotted timeframe. Students discussed that it was hard to capture the whole restaurant experience in such a short amount of time. Some students frequently found themselves in a high-pressure time crunch to perform their responsibilities. One student said, “It’s hard because you want to take your time and do well, but you also have to learn to work quickly and have things done on time, which I felt was challenging” (participant 6, dietetics). With a few exceptions, the general feeling among the students was that SOR participation was a valuable investment of their time.

Opinions on the role of nutrition within SORs varied widely among the students. Of note is that none of the students discussed nutrition as a part of their experience until prompted by the interviewer. Most students felt that nutrition was not involved to a significant degree. Some students were satisfied with this because they felt the purpose of the class was not to promote nutrition, but rather to provide foodservice management experience. One student explained, “The purpose is to show us how management works in a food service organization and to be a part of it” (participant 12, dietetics). Nonetheless, some students felt that nutrition was sufficiently incorporated because a variety of food and food groups were present in the meals prepared. Other students were disappointed with the limited involvement of nutrition and felt that it should play a bigger role in the course. A few students mentioned that customers would often ask if the meals were healthy. One student observed,

“I think my dietetics background and education has tuned my brain into thinking that that’s how we need to eat and to serve that population. Especially when we are, as a dietetics class are running the [SOR name], I felt like people would even ask, is this just super healthy? And I’d feel like I had to say no, not really. It’s just normal food. Kind of disappointed in us as [SOR name.] I felt like we could have done better” (participant 3, dietetics).

### 3.3. Theme 3: Moving Forward, Added Value, and Growth

Within the third theme, Moving Forward, Added Value, and Growth, two sub-themes were identified. These are benefit to student and career influence. Regarding benefit to student, it was discussed that students gained more from the course than anticipated. They grew in areas such as communication, leadership, and customer interaction. They also reported a greater ability of problem-solving and ability to work under pressure. Many students noted that their confidence increased throughout their SOR experiences. They felt that the confidence and experience they gained would benefit their personal lives as well as their careers. One student said, “I think at first, the course, I was like, whoa, this is very daunting. I’m a little nervous about it. But then once I started doing it, I gained a lot more confidence, and I was able to see that this is something I can learn a lot out of” (participant 2, dietetics).

The second sub-theme was career influence. Students felt that SORs prepared them for future careers. For students pursuing managerial roles, the SOR experience closely paralleled their future career environment. For those pursuing other positions, such as clinical or community nutrition, SORs helped them become more well-rounded and better qualified to work in various settings. One student said, “I am still not entirely sure where I want to go with dietetics. But I do recognize the importance of food management and food service management in the field. Whether or not I am a food service manager, I think that there are always times that we deal with food, whether it’s in a work setting or in events or whatever it is” (participant 11, dietetics).

SORs also influenced students’ career decisions. Some students found that they became more interested in foodservice management through their SOR participation. Conversely, some discovered that they were less interested in foodservice management which pushed them to pursue other areas of dietetics or hospitality. One student said, “Going into this class, I hadn’t had a lot of hospitality or food service experience. And coming out of it, I think I would really like enjoy the idea of being a food service manager, working in that type of setting just because I hadn’t really considered it before” (participant 18, dietetics).

### 3.4. Skills

During the interviews, participants were asked what foodservice skills and management skills they gained through their SOR experiences. The foodservice skills mentioned generally fell under the domains of cooking, safety, service, food preparation, and equipment use. Management skills included domains of interpersonal, organizational, procedural, and personal. Table 3 indicates the number of references made in each of these categories.

In general, students felt that the foodservice and management skills they gained would be of benefit to them in the future. This view was challenged by some who mentioned that they did not expect to use some of the foodservice skills directly in their future positions. However, most students felt that understanding in this area will be important as they work as part of an interprofessional team. Many felt that the management skills they learned will be beneficial regardless of their future roles. One student said, “I feel like any management skill I accrue at any point in my life will definitely help me in my future career” (participant 9, hospitality).

## 4. Discussion

Many research studies have been conducted previously on the use of experiential learning in higher education settings. Results have indicated that when participating in experiential learning students appreciated the opportunity to apply concepts and felt the experience was beneficial [25,26], retained information better [27], and were able to apply the concepts in a real-world setting [25]. These results are similar to the results of this current study which also indicated that students viewed their SOR experience positively and felt it was beneficial to their future careers. However, this study is unique because it extensively explored the experiences of both dietetic and hospitality students participating in an immersive experiential learning program, the SOR (as detailed in the results above and discussion below). This area has not been studied in previous research.

Thematic analysis of interview data revealed three overarching themes related to students’ relationships with fellow students and instructors, their various experiences in the SOR, and the value they placed on their SOR experience. Specifically, students found interpersonal interaction to be an integral part of their SOR experiences. Working together with peers increased confidence and performance in assigned tasks and improved communication throughout the kitchen. In addition to improving performance, working together also facilitated relationships among peers and instructors. Chung et al. [28] showed the value of relationships in the workplace in a study which indicated that groups composed of friends perform better than groups not composed of friends. Ji and Yan et al. [29] further supported the value of teamwork by showing that team structure is positively associated with team performance and coordination. The ability to work as a high-functioning team is a critical skill for foodservice managers since they often coordinate with professionals across many disciplines including hospital administrators, restaurant managers, marketing specialists, dietitians, and foodservice workers. Interpersonal skills are also necessary for dietitians who work in direct patient care or in management capacities [30]. SORs provide an opportunity for students to practice teamwork which can prepare them to navigate the multidisciplinary fields of foodservice and dietetics.

SOR instructors gave their students a degree of independence in completing their tasks. Students felt that this autonomy helped them learn to work under pressure, solve problems, and make decisions. These skills too are valuable for foodservice managers and dietitians who must manage a wide range of pressing responsibilities [15].

The hands-on approach of SORs was beneficial for students. Research shows that experience-based education leads to greater competency in learning outcomes for foodservice and dietetics programs [31]. Students feel that experiential learning promotes better understanding, creates an optimal environment for applying theory, and encourages critical thinking [32]. Additionally, including experience-based learning in hospitality programs helps students develop management skills, such as leadership, financial management, and understanding of how organizations function [33]. SORs provide a setting for experiential learning which can prepare students to meet the demands of their various careers.

Students noted that SORs created a time crunch in which they were under pressure to complete their required tasks in the allotted time. Research by McCord and Matusovich [34] shows that learning under a time crunch can cause learning to become secondary to completing the required tasks. Less time constraints allow for deeper learning and internalization. However, although a pressured environment may not be the ideal setting for education, it may benefit students to learn to work efficiently in a time crunch since foodservice managers are often required to do so. Instructors of SORs should ensure that experience-based performance outcomes are met without sacrificing other competencies. Instructors should also work to strike an appropriate balance between providing a realistic foodservice management experience (pressured environment) and ensuring the environment is appropriate for student learning.

Most students felt that nutrition did not receive a significant focus in their SORs, but opinions differed concerning the adequacy of nutrition’s role even across majors. Some felt that nutrition was not meant to be a focal learning outcome of SORs, so they were satisfied with its minimal inclusion. Others indicated that a greater emphasis on nutrition would be a significant affordance to their education. Mathews et al. [19] explored how SOR managers integrate nutrition principles into their programs and found that nutrition principles are taught in some programs but are not typically emphasized as a major component. Mitchell et al. [35] found that nutritional competency is important for hospitality students, but its place is often undervalued in education. The lack of nutrition in education may perpetuate its lack of perceived importance in the restaurant industry [8]. Some instructors have shown the potential for nutritional focus in SORs by including parameters such as caloric limits, sodium restrictions, fiber requirements, and alternate diet options [16]. Instructors should consider how integration of nutrition into SORs could equip students to better meet the nutritional demands of the industry and society.

SORs were reported to better qualify students for their future careers. Research by Cranmer [36] indicated that although college graduates go through extensive learning in the classroom, this instruction does not necessarily equip them with the skills they need in the workplace. Cranmer [36] also found that when work experience was added to education, students had increased ability to find graduate-level jobs immediately or shortly following graduation. SORs provide work experience that may open more job opportunities for foodservice managers and increase their capacity to contribute in the workforce.

## 5. Conclusions

Engaging in experience-based learning through SORs helped students develop meaningful relationships with other students and have positive mentoring experiences with instructors. These interactions fostered interpersonal growth, teamwork opportunities, and leadership skills. Through this hands-on approach students also gained practical foodservice and management skills as well as a comprehensive grasp of systems operations. Finally, the SOR experience helped students feel better prepared for their future careers, and even helped some to make career decisions. Education programs should continue to foster experiential learning opportunities through use of the SOR.

Results indicated that although some students felt that nutrition principles were effectively addressed during their SOR experience, many students acknowledged a lack of nutritional emphasis in their programs and expressed a desire for greater application of the nutritional principles taught in other classes. Foodservice and/or management educators should consider adjusting SOR curriculum to incorporate more nutrition principles. Improving the nutrition of SOR menu items could help students learn how to prepare and market nutritious menu options to customers. This could better prepare students to improve public health among a population which is increasingly eating outside food.

SORs are an experiential learning tool used in some dietetics and hospitality programs across the country. Although these lab experiences provide a real-life hands-on learning environment for students, they require substantial resources to operate including space (fully furnished commercial style kitchen and dining room), supplies, faculty and staff, and an operating budget. Understanding students’ perceptions of their SOR experience is critical in helping programs ensure that investment required to operate the labs is justified and in identifying ways to improve the use of SORs. Results of this study indicate that students view their experiences in SORs favorably and thus it provides a justification for continued use of SORs in the future.

This study included students from both hospitality and dietetics programs to offer a comprehensive view of students’ perceptions. The sample size was adequate for the exploratory purposes of this study and the findings can inform future SOR student-focused quantitative studies. A limitation of this study is that we did not know how the SOR course was taught and managed in each program. Moreover, the level to which the managing faculty focus on nutrition and/or management aspects of the SOR could influence students’ perceptions of their experience. Future research should focus on identifying whether or not increasing the use of nutrition curriculum in SORs improves students’ perceptions of their SOR experience.

## Figures and Tables

**Table 1:** Demographic characteristics of participants (n = 18).

Characteristic	n (%)
University ^a^	
Brigham Young University	9 (50.0)
Iowa State University	6 (33.3)
Kansas State University	2 (11.1)
Eastern Kentucky University	1 (5.6)
Participation in Student-Operated Restaurants ^a^	
Currently participating	9 (50.0)
Previously Participated	5 (27.8)
Have not participated but will participate in the future	4 (22.2)
Educational Program	
Dietetics	13 (72.2)
Hospitality	5 (27.8)
Year in School ^a^	
Junior	9 (50.0)
Senior	8 (44.4)
Other	1 (5.6)
Age	
20	2 (11.1)
21	5 (27.8)
22	5 (27.8)
23	3 (16.7)
24	2 (11.1)
25	1 (5.6)
Race/Ethnicity ^a,b^	
White	14 (77.8)
Hispanic or Latino	3 (16.7)
Asian	2 (11.1)
Gender	
Female	15 (83.3)
Male	3 (16.7)

^a^ One or more option items had zero respondents and were not included in the table; ^b^ Respondents could check all that apply, total may exceed 100%.

**Table 2:** Illustrative quotes for each theme and sub-theme mentoring.

**Theme 1: Interpersonal Relationships and Mentoring**	
Sub-theme 1: Working together	“I think I like the team aspect the most and the people I get to work with” (Participant 10, SOR 1, Dietetics). “I think we made a really good team. We saw each other three times a week in person, which none of my other courses met in person” (Participant 15, SOR 2, Hospitality). “Like I said, the attitude that everybody had, we were all in it together and we were all willing to help each other” (Participant 18, SOR 3, Dietetics).
Sub-theme 2: Relationships	“I think it helped me understand people and get to know them better too because there were group projects that we’d worked on before, which is still good, but it’s just a different context” (Participant 1, SOR 1, Dietetics). “Well, you’ll be interacting with people every day where they’re the professors or the interns or customers who come in or just your co-workers. And so you interact with them all on a different level and it’s more casual…” (Participant 7, SOR 1, Dietetics) “I just thought the relationships that we built in the kitchen, we had a really tight knit group of people that were in there. So it was really like a team feel. And I made a lot of friendships working there…” (Participant 18, SOR 3, Dietetics)
Sub-theme 3: Mentoring	“I really like the independence that we’re given…The interns and the teachers, they come around and kind of explain what we’re going to be making and kind of give some details, but other than that, we’re pretty much on our own to figure things out. And I really like that because I feel that’s another way that I felt more confident in the class. It’s like, ‘Oh, wow, I can really do this. This is something I can do on my own or with somebody’s help.’ I really like that” (Participant 6, SOR 1, Dietetics). “We could work one-on-one with our instructor to work on recipes or to learn other things like that” (Participant 9, SOR 2 Hospitality). “…our instructors were awesome. They were super helpful and they answered all questions and they’d been through this before so they were really understanding about how it’s stressful and nerve wracking at the beginning and mistakes are going to get made, but they were really good at being on top of it and making sure they set us up for success while also letting us be independent” (Participant 18, SOR 3, Dietetics).
**Theme 2: Assessment of Immediate Experience**	
Sub-theme 1: Hands-on Learning	“I think it will be very crucial to our understanding and help us develop skills we learned in the classroom because I’m a very hands-on learner” (Participant 5, SOR 1, Dietetics). “…It was kind of nice to get that experience because it’s like none of my other classes, I’ve taken culinary labs before. And in those courses, you do the same thing every day. Whereas here it’s kind of like you’re stepping into the kitchen and you’re like, “Okay, this is a completely new job. It’s a completely different job. It’s a completely new experience.” So that’s kind of cool. And I think that’s the best part of the class that I get to experience every single role” (Participant 14, SOR 3, Hospitality). “I think I probably actually liked the diversity of the different positions we did just because you got to experience every part of the kitchen and also just having that final kitchen manager day and being able to step back and look at everything, not necessarily from an outsider’s perspective, but just like being able to see the organization as a whole was probably my favorite part. Just to see how all the different, I guess you could say, wheels came together to make the organization run smoothly” (Participant 16, SOR 3, Dietetics).
Sub-theme 2: Time	“I would say probably the most challenging thing was that it takes up such a large chunk of your day” (Participant 2, SOR 4, Dietetics). “I think what’s most challenging about [SOR name] is the fact that you’re running this little mini restaurant in three hours. So our lab was 10:30 to 1:20, so you’re doing the opening tasks and you’re doing a service and then you’re doing closing tasks all within three hours, which is kind of weird. So that was kind of challenging because just at the end of the day, it felt like a lot of stuff sometimes just wouldn’t get done” (Participant 9, SOR 2, Hospitality). “It seemed like a lot, but when I was there it never really did seem that bad. The time always flew and I always enjoyed my time there… Sometimes it did feel like it took a lot of time when I could be doing homework or something, but generally I felt like the time was well used and well spent” (Participant 10, SOR 1, Dietetics).
Sub-theme 3: Nutrition	“Well, they did always kind of make the joke that, “None of this food is super healthy” (Participant 2, SOR 4, Dietetics). “…I do think it’s interesting, maybe a little ironic that we’re dietetic students, but we don’t create meals that are on the healthier side of things. But that they’re full of margarine and heavy cream. And again, we’re making really yummy meals. So I know that’s the main purpose of Pen Court, and to teach students the role of food management and just food organizations. But I do think it’s a little... There’s a lack of nutritional influence on the meals that we make” (Participant 4, SOR 1, Dietetics). “I feel really good about it. I feel like it’s very balanced with the message I think that our dietitians are generally trying to send is that we’re not food police. We’re there to make sure you get all of the nutrients and are happy with it” (Participant 10, SOR 1, Dietetics). “I wasn’t like upset that we didn’t go over it, but I think it would be a good addition to the course” (15, SOR 2, Hospitality). “We really didn’t have a whole lot of nutrition sort of curriculum. I would say we didn’t, it wasn’t something that we really focused on. It was more so just focusing on management food service” (18, SOR 3, Dietetics).
**Theme 3: Moving Forward, Added Value, and Growth**	
Sub-theme 1: Added Value	“…I feel like they’re valuable in a couple of different ways, both in things with my family, being able to use those, but as well as taking it into future classes and future careers, being able to apply the things I’ve learned here. And to be able to get things done productively, quickly, help and work stronger in a team, and to make sure it’s all presentable and safe” (Participant 10, SOR 1, Dietetics). “Everywhere has a manager, even if that’s not their specific title, but I definitely think it’s important whether you’re just a leader in your department or whatever it looks like they’re very useful” (Participant 15, SOR 2, Hospitality). “It was just a lot more fun and a lot more relevant to me than I thought that it was going to be…it is a really difficult class, but at the end of the day, you get in what you put out. And I think that’s kind of the whole idea of it” (Participant 18, SOR 3, Dietetics).
Sub-theme 2: Influence on Career	“…Being a dietitian is really cool because there’s so many different disciplines you can go into. And even though food production management or food production or the food industries aren’t my primary choice, I’m more leaning towards clinical or sports. You never really know what might be required of your situation… even though this isn’t my primary discipline that I want a primarily focus, I know it still spills into it like other disciplines” (Participant 5, SOR 1, Dietetics). “…The confidence that I gained from the class it made me feel like, “Oh, this is something I could potentially do.” And, it’s made it more interesting than it was before” (Participant 6, SOR 1, Dietetics). “I feel it’s always been in the back of my mind that I’ve wanted to start out as some sort of management after I graduated, but having the experience now, I feel it’s pushing me more towards that management role” (Participant 8, SOR 3, Hospitality).

**Table 3:** Categorization of foodservice and management skills learned by participants.

Foodservice Skills		Management Skills	
Skill Category ^a^	n ^b^	Skill Category ^a^	n ^b^
Cooking	25	Interpersonal	28
Safety	19	Organizational	14
Service	13	Procedural	14
Food Prep	9	Personal	8
Equipment Use	6		

^a^ Skill categories were developed by research to more clearly and succinctly represent the variety of skills mentioned by participants; ^b^ n represents the total number of participants (excluding those who had not yet participated in an SOR) who mentioned a skill related to each specific skill category.

## Data Availability

The data presented in this study are available on request from the corresponding author. The data are not publicly available due to the current IRB approval restrictions.
